# Regioselective Radical Alkylation of Arenes Using Evolved Photoenzymes

**DOI:** 10.21203/rs.3.rs-2602958/v1

**Published:** 2023-02-23

**Authors:** Claire G. Page, Jingzhe Cao, Daniel G. Oblinsky, Samantha N. MacMillan, Shiva Dahagam, Ruth M. Lloyd, Simon J. Charnock, Gregory D. Scholes, Todd K. Hyster

**Affiliations:** 1 Department of Chemistry and Chemical Biology, Cornell University, Ithaca, New York 14850, United States; 2 Department of Chemistry, Princeton University, Princeton, New Jersey 08544, United States; 3 Prozomix. Building 4, West End Ind. Estate, Haltwhistle (UK)

## Abstract

Substituted arenes are ubiquitous in molecules with medicinal functions, making their synthesis a critical consideration when designing synthetic routes.^1,2^ Regioselective C–H functionalization reactions are attractive for preparing alkylated arenes,^3–5^ however, the selectivity of existing methods is modest and primarily governed by substrate electronic properties.^6,7^ Here, we demonstrate a biocatalyst-controlled method for the regioselective alkylation of electron-rich and electron-deficient heteroarenes. Starting from an unselective ‘ene’-reductase (ERED) (GluER-T36A), we evolved a variant that selectively alkylates the C4 position of indole, an elusive position using prior technologies. Mechanistic studies across the evolutionary series indicate that changes to the protein active site alter the electronic character of the charge transfer (CT) complex responsible for radical formation. This resulted in a variant with a significant degree of ground state change transfer in the CT complex. Mechanistic studies on a C2 selective ERED suggest that the evolution of GluER-T36A helps disfavor a competing mechanistic pathway. Additional protein engineering campaigns were carried out for a C8 selective quinoline alkylation. This study highlights the opportunity to use enzymes for regioselective reactions where small molecule catalysts struggle to alter selectivity.

Alkylated arenes are essential for the structure and function of nearly all modern pharmaceuticals and agrochemicals. Consequently, countless cross-coupling methods have been developed to alkylate arenes, where a halogen or organometallic substituent’s position determines the transformation’s selectivity.^1,8,9^ As these reactions require pre-activation of the arene, a more streamlined approach is to alkylate unactivated arenes.^10,11^ The most common examples of this approach are transition metal-catalyzed and Friedel-Crafts type alkylations.^3,4^ In these reactions, the regioselectivity of alkylation is substrate-controlled and based on the arene and alkylating agent’s inherent electronic and steric properties.^5,12,13^ While this can result in single regioisomers where alkylation only occurs at the most activated position, more often, the steric and electronic effects are subtle and result in mixtures of regioisomers (Figure 1A). We imagined that by developing a catalyst-controlled alkylation strategy, we could overcome the inherent reactivity of the substrate and enable alkylation at positions that are elusive to prior methods.

The advent of photoredox catalysis and electrosynthesis has spurred the development of radical-mediated arene alkylation reactions.^7,14,15^ The regioselectivity of these reactions is governed by the “*philicity*” of the alkylating radical and the electronic and steric characteristics of the arene.^6^ While these factors can result in selective alkylation at a single position, unbiased arenes often form mixtures of regioisomers. Phipps and coworkers recently demonstrated that chiral phosphoric acids could deliver *α*-amino and *α*-oxy radicals selectively to the C2 position of pyridines, disfavoring the formation of the C4 isomer.^16,17^ Alternative catalytic strategies for controlling the regioselectivity of radical arene alkylation remain elusive.

Enzymes are attractive catalysts for transformations requiring chemo-, regio-, and enantioselectivity due to their precise control over conformation and electronics. However, they are not viewed as a panacea for selectivity challenges because they are perceived only to catalyze their native reactivity. Our group has developed a set of electron transfer mechanisms to enable natural oxidoreductases to catalyze non-natural reactions.^18–22^ In particular, flavin-dependent ‘ene’-reductases (EREDs) can catalyze intermolecular reductive couplings of alkyl halides with alkenes or nitronates to provide the coupled product in good yield with negligible hydrodehalogenation of the starting material. High fidelity for the intermolecular coupling is possible because radical formation occurs via photoexcitation of an enzyme-templated ternary charge-transfer complex between the alkyl halide, SOMOphile, and flavin hydroquinone (FMN_hq_), ensuring that radical formation only occurs when both substrates are present within the protein active site.^23^ As the spatial orientation of the two coupling partners is vital for CT complex formation, we questioned whether this feature could be exploited to control the regioselectivity of arene alkylation. By engineering the protein, we could reorient the alkyl halide over the desired position for alkylation (Figure 1B).

We began by investigating the alkylation of indole because of its prevalence in bioactive molecules and the possibility of alkylation at several positions. Friedel-Crafts alkylations of indole typically occur at the C3 position while reactions involving radical alkylating agents favor the C2 position.^24,25^ Indeed, under photoredox conditions, indole alkylation by *α*-chlorodimethylacetamide **1** occurs in 47% yield with 4:1 selectivity favoring the C2 position over other regioisomers (Supplemental Figure 1).^26^ We screened a panel of EREDs and found two with divergent selectivity (Supplemental Table 1). AspER, an ERED from *Aspergillus nidulans*, alkylates the C2 position in 48% yield with trace amounts of other regioisomers while GluER-T36A catalyzed the reaction in 83% yield and provides product in a [1:2 ratio of C4 : (C2+C3)] regioisomers (Figure 2A). As methods for selectively alkylating at the C4 position of indole are rare, we initiated a protein engineering campaign to create a C4 selective variant of GluER-T36A.^27^

We began by screening the reaction against 24 GluER-T36A variants our lab had previously developed which had mutations at five positions thought to interact with the substrate.^23^ GluER-T36A-Y343F emerged as the most promising variant as it decreased the formation of the C3 product in favor of the C2 and C4 regioisomers (94% yield [1:1.5 C4:(C3+C2)]) (Supplemental Figure 2). Optimization of the reaction conditions using this variant revealed that increasing the NADP^+^ loading from 1 mol % to 5 mol % increased the ratio of C4 and C2 adducts with only a slight decrease in overall yield (73% yield [1:1.2 C4:(C3+C2)]), Supplemental Table 2). With an improved catalyst and reaction conditions in hand, we conducted iterative site saturation mutagenesis on residues surrounding the protein active site that were not represented in the original library (Figure 2B). After five rounds, we identified GluER-T36A-Y343F-T25L-T231V-G270M-Q232W-T268D (referred to as PagER) which affords the C4 regioisomer in 90% yield with less than 10% yield of C2 and C3 product [9:1 C4:(C3+C2)]. Optimization with the penultimate mutant showed switching the buffer from tris(hydroxymethyl)aminomethane (Tris) buffer to 100 mM potassium phosphate (KP_i_) buffer improved the regioselectivity (Supplemental Table 3). Further optimization with PagER demonstrated that glucose loading could be lowered to 0.5 equivalents with no loss in yield or regioselectivity (Supplemental Table 4). An X-ray crystal structure was collected for PagER (8FW1) which indicates the overall fold of the protein remains largely unchanged despite the mutations (Supplemental Figure 56).

With C2 and C4 selective variants in hand, we conducted a series of experiments to determine the mechanism of radical initiation for each enzyme. To systematically probe which oxidation state is responsible for radical initiation, we conducted a series of control experiments. When AspER was reduced with 40 equivalents (by comparison to enzyme) of NADPH and then supplied with substrate and allowed to stir in the dark for 24 hours, no product was observed confirming that ground state FMN_hq_ cannot initiate the reaction (Figure 3A). Next, we attempted fluorescence quenching studies with FMN_hq_ and the substrates to determine whether excited state FMN_hq_ is responsible for the observed reactivity, however, the steady-state fluorescence of FMN_hq_ could not be measured due to quenching by the protein. We shifted to ultrafast transient absorption spectroscopy to better understand possible excited state dynamics. When FMN_hq_ was excited, a tri-exponential decay is observed with excited state lifetimes of 3 ps, 42 ps, and 475 ps (Supplemental Figure 31). The lifetime of FMN_hq_ in AspER is significantly shorter than what was previously observed with GluER variants, suggesting the residues in AspER are better able to quench the excited state.^18,19^ Addition of either chloroamide **1** or indole alone did not alter the excited state lifetimes (Supplemental Figure 33 and 37). However, when both substates were added to the reduced enzyme, excitation led to the immediate formation of the neutral semiquinone (FMN_sq_H). This species decays to the FMN_hq_ in 21 ps suggesting that mesolytic cleavage, C–C bond formation, and oxidation of the radical occur on this timescale (Figure 3B, Supplemental Figure 35). This study indicates that radical initiation can occur from the excited FMN_hq_ and is terminated by back electron transfer to FMN_sq_H since no long-lived excited state absorptions of FMN_hq_, FMN_sq_^−^, or FMN_sq_H are observed. This is likely through an enzyme-templated ternary CT state complex.

We then attempted to find a CT state with AspER. When reduced with excess sodium dithionite, a single electron reductant known to completely reduce FMN to FMN_hq_ in many EREDs, we observe incomplete reduction of AspER resulting in a mixture of FMN_hq_ and anionic semiquinone (FMN_sq_^−•^) (Supplemental Figure 16). Addition of chloroamide and indole led to the growth of a weakly absorbing spectral feature centered at 500 nm, however, it was ambiguous whether the feature was indicative of a CT complex or a flavin redox event. Attempts to repeat this experiment with NADPH also showed NADPH could not completely reduce the protein but showed a much larger change in spectral features centered around 500 nm (Supplemental Figure 17). This combined with the transient absorption data suggests AspER can also template a charge transfer state between the FMN_hq,_ the chloroamide, and the indole to initiate the radical.

As the CT complex in AspER was difficult to visualize using steady-state UV-Vis absorption, we hypothesized that a secondary pathway could be operative with AspER. In a previous report, we found that FMN_sq_^−•^ could initiate a radical cyclization to afford oxindoles.^28^ This oxidation state was formed when the enzyme was irradiated with visible light in the presence of a buffer that can function as a single electron reductant.^28^ Based on this example, we questioned whether a similar mechanism was operative for indole alkylation. During reaction optimization, we found that the coupling reaction occurs without turnover mix in tris(hydroxymethyl)aminomethane (Tris) buffer (100 mM, pH = 9.0), a good buffer for single electron reductions. EPR spectroscopy confirmed that FMN_sq_^−•^ is formed when AspER is irradiated in Tris buffer (Supplementary Figure 40). In contrast, buffers composed of poor single electron reductants, like phosphate, provided product in 7% yield under otherwise identical reaction conditions. This result, when coupled with our FMN_hq_ control experiments, suggest that FMN_sq_^−•^ could initiate this reaction. To further confirm that FMN_sq_^−•^ can initiate the reaction, we titrated AspER with sodium dithionite until a UV-vis spectrum consistent with a mixture of FMN_ox_, FMN_hq_ and FMN_sq_^−•^ was formed (Supplemental Figure 42). When substrate is added under anaerobic conditions and left to sit in the dark, 2% yield of C2 product is formed (Figure 3C). These results indicate that ground state FMN_sq_^−•^ can initiate radical formation. As initiation from FMN_sq_^−•^ does not require a charge transfer complex, it is possible that the selectivity of this enzyme is simply following the substrate’s inherent selectivity. Indeed, when GluER-T36A is reduced to the FMN_sq_^−•^ using sodium dithionite, the C2 product is now formed preferentially with the same ratio observed when turnover mix is removed in Tris buffer ([1.3 :1 C2:(C3+C4)] (Figure 3D and Supplemental Figure 43). Based on this data, we hypothesize that initiation through the FMN_sq_^−•^ results in more C2 product formation, largely regardless of the enzyme.

From the transient absorption results and the ground state results, we conclude that AspER, has access to two different pathways for initiating the radical from the *α*-chloroamide (Figure 4E). Based on the significantly short time scale of the mesolytic cleavage and C–C bond formation, it is likely the enzyme binds both the indole and the *α*-chloroamide in a highly preorganized matter. This pre-organization could assist in ensuring coupling occurs over hydrogen atom transfer to the *α*-acyl radical when the *α*-chloroamide is reduced by the ground state semiquinone. Due to the fact AspER performs the best with no external reductant and requires buffers that can act as single reductants, it is likely the CT pathway is inefficient and the semiquinone pathway is the predominant pathway under the optimal reaction conditions. The inefficiency of the CT state is further supported by the inability to fully reduce the protein and the weak spectral changes observed when the substrates are added to the reduced enzymes. The presence of the semiquinone pathway in GluER T36A has deleterious effects in producing the C4 product, which explains why an external 2 electron reductant like NADPH is needed to achieve good yields of the C4 product.

We then conducted experiments to better understand the reactivity of PagER. As the C4 selectivity of this enzyme contrasts with the inherent selectivity of the substrate, we hypothesized that PagER was better at templating a ternary CT complex between the alkyl chloride, indole, and FMN_hq_ in the correct orientation for C4 alkylation compared to GluER-T36A. To investigate this hypothesis, we conducted UV-Vis spectroscopy with each mutant in search of changes to a hypothesized CT state. We initially reduced all mutants with sodium dithionite to form FMN_hq_, which has negligible absorption above 400 nm in all cases (Supplemental Figures 18–27). Next, we added chloroamide **1** to the reduced enzyme and observe a weakly absorbing CT complex with maximum absorption **λ**_max_ ~ 490 nm for all mutants (Supplemental Figures 18, 19, 21–27). Finally, indole was added to the samples containing the reduced enzyme and chloroamide **1**. With GluERT36A, the overall spectral feature of the CT complex was largely unchanged, with only a small growth in the absorption around 490 nm (Figure 4A, green trace). In contrast, PagER’s spectral feature was significantly altered by the presence of indole with new absorption features at 370 nm, 400 nm, and 480 nm (Figure 4B, green trace); a spectral feature that closely resembles FMN_sq_^−•^ in old yellow enzyme 1.^29^ When this solution was allowed to stand for 5 minutes, we observe a decrease in dithionite absorption (317 nm) and the growth of a spectral feature indicative of oxidized FMN (Figure 4B, red trace). This solution was then filtered through a 10 kDa MW filter to remove the small molecules, oxidized FMN and FMN_hq_ are recovered. This result suggests that the observed spectral feature is not, in fact, a charge transfer complex and is instead indicative of a ground state electron transfer event (Figure 4B and Supplemental Figure 18). To determine whether PagER can facilitate electron transfer without photoexcitation, we ran a no-light control with PagER and found the coupled product in 16% yield [7:1 C4:(C2+C3)] (Figure 4C). In contrast, only unreacted starting material was recovered when the same experiment was run with GluER-T36A. Repeating this control reaction with every mutant in the evolution campaign showed this ground state reactivity only appears after the introduction of the Q232W mutation in the fifth round of engineering (Supplemental Figure 25, 26 and Supplemental Table 6).

These combined mechanistic experiments implicate there were significant changes in the characteristics of the charge transfer complex over the evolutionary campaign. As described by Mulliken, the wavefunction for ground state CT complex can be written as a linear combination of the “non-bonded” component and the ionic complex.^30, 31^ In our system, we can describe the ground state and excited state wavefunction using the following simplified equations:

$$\psi_{GS}=a\left(\psi_{FMN(HQ),R-Cl,\text{Indole}}\right)+b\left(\psi_{FMN(SQ),R-Cl^{\cdot -},\text{Indole}}\right)$$

$$\psi_{ES}=b\left(\psi_{FMN(SQ),R-Cl^{\cdot -},\text{Indole}}\right)-a\left(\psi_{FMN(HQ),R-Cl,\text{Indole}}\right)$$

Using this equation, the ratio of the constants (*q* = *b/a*) describes the degree of ground state charge transfer (*q*). Based on experimental observation, we hypothesize that the *q* is very small (*a* >> *b*) with GluER-T36A thus accounting for the lack of ground state reactivity with this variant. PagER, however, has more charge transfer in the ground state (*q*_*PagER*_ > *q*_*GluER−T36A*_). As mesolytic cleavage of the C–Cl bond in the charge transferred component is irreversible and leads to product, enzymes with higher *q* values should display dark reactivity.^32^ We hypothesize that photoexcitation is still required for high yields because the excited state has a higher degree of electron density localized on the chloroamide^33,30^

The energy gap of the electron transition from the *ψ*_*GS*_ and *ψ*_*ES*_ can be described using the following equation:

$$hv_{CT}=IP-EA+\omega$$

where IP = ionization potential of the donor; EA = electron affinity of the acceptor; and *ω* is the Columbic attraction between the charged species.

In this equation, the ionization potential of the donor and electron affinity of the acceptor can be approximated by the redox potentials of substrates. The Coulombic term can be approximated to be $\omega =-\frac{e^{2}}{r_{DA}}$ and describes the interactions between the two molecules (electrostatics, Van der Waal repulsion etc.) where *r*_*DA*_ describes the distance between the donor and acceptor molecules. This term is sensitive to steric and electronic changes to the acceptor or donor molecules as demonstrated by work done to tune the band gap in organic semiconductors.^34,35^ The flavin redox potential can be approximated using a method described by Massey which uses the energy of the CT complex formed between *p*-methoxyphenol and FMN_ox_ to determine the redox potential.^29^ When looking at these potentials across the evolutionary series, we see that the protein engineering campaign has resulted in a flavin cofactor that is roughly 100 mV more reducing than the parent (Supplemental Figure 49, 50). This can be correlated to a lower ionization potential for the FMN_hq_.^36^ The primary mutation contributing to this change is T25L. This residue interacts with the N5 position on the flavin cofactor and presumably decreases the stability of the flavin semiquinone. Indeed, Massey showed in OYE1 that when the homologous threonine is mutated to a hydrophobic residue (alanine), OYE1 T37A’s semiquinone was significantly destabilized through loss of a hydrogen bonding interaction to flavin.^37^ This mutation is beneficial in PagER by preventing access to the semiquinone in solution, thereby decreasing the amount of C2 product formed through the semiquinone pathway. When comparing photoreduction rates, PagER is much slower to be photoreduced in 100 mM Tris pH 9 buffer and appears to go straight to the FMN_hq_ while GluER-T36A forms some of the semiquinone (Supplemental Figures 51 and 52). When the leucine is mutated back to threonine (PagER-L25T), we see an overall decreased yield with the C2 product formed as the major product (21% yield [1:1.3 C4:(C3+C2)]).

Overall, these studies indicate the importance of the T25L mutation in dictating the selectivity by tuning the electronics of flavin and eliminating access to the semiquinone in solution. We believe that the additional mutations around the protein active site primarily impact the columbic term of the CT complex and suggest that mutations are decreasing the distance between the donor and acceptor molecules. This impacts selectivity by orienting the arene in the desired position for alkylation. Additionally, we expect that a more closely packed CT complex should also result in stronger hyperconjugative interactions between the chloroamide and indole, increasing the electron affinity of the electron acceptor by making the reduction potential of the chloroamide more positive. Moreover, these strengthened interactions should also result in a high degree of charge transfer in the ground state, consistent with our previous results using *α*-bromoacetophenone as the radical precursor for ground state radical hydroalkylations.^38^

An interesting implication of changes over the evolutionary series is that the FMN_sq_^−•^ becomes more reducing making oxidation of the radical intermediate more thermodynamically challenging. To probe whether this impacts the rate determining step, we conducted initial rate kinetics with D4/H4 indole and looked for a kinetic isotope effect (Supplemental Figure 54). We were intrigued to find a normal secondary kinetic isotope effect of 1.2, indicative of hyperconjugative effects of the C4-H/D bond in the rate determining step (Figure 4D). This suggests the rearomatization step still goes through the Wheland intermediate and the electron transfer from the indoyl radical to the FMN_sq_^−•^ is rate limiting.

With mechanistic understanding in hand, we then explored the substrate scope of the reaction with PagER. The enzyme maintains excellent regioselectivity across an array of *α*-chloroamides, including the corresponding Weinreb amide, azetidine amide, and *α*,*α*,*α*-chlorodifluorodimethylacetamide. In the latter case, alkylation only occurs at the C4 position. The enzyme was also tolerant to various substituents around the aromatic ring. *N*-methyl indole is alkylated at the C4 position, indicating that the N–H bond is not required for high selectivity. Halogenation around the arene resulted in altered selectivity. 5-Bromoindole was alkylated at the C6-position in good yield and modest regioselectivity while 6-bromoindole was alkylated at the C7-position. Beyond indoles, *N*-methylaniline could be alkylated selectively at the *para*-position (10:1 selectivity) using GluER-T36A-Y343F-T25L in 67% yield. Importantly, this *C*-alkylation outcompetes background N-alkylation which forms in 46% yield without enzyme (Supplemental Figure 14). The reaction was run on preparative scale with lyophilized lysate to afford pure C4 product in 42% yield. Finally, the C4-alkylation product can be derivatized. Under basic conditions, the amide was hydrolyzed to the corresponding carboxylic acid. Using photoredox conditions developed by Nicewicz, the carboxylic acid could be converted to the corresponding methyl substituted arene (Supplemental Figure 15).^39^

Next, we tested whether this alkylation method could be used with electron-deficient arenes and began testing quinoline heterocycles. Radical addition to quinoline typically occurs at the C2 or C4 positions.^7,40^ Radical methods for alkylating the arene are elusive, with transition metal catalysis alkylations relying on directing groups or the templation of a catalyst to the nitrogen.^41,42^ We hypothesized that our method would favor alkylation on the more electron-rich arene ring.

Screening quinoline across a panel of EREDs showed that alkylation occurs at the C4, C5 and C8 positions. GluER-T36A-Y343F-T35L was identified as the optimal catalyst as this enzyme is very selective for the C8 position, albeit with lower overall yield. Further screening of the mutants identified during the indole campaign identified GluER-T36A-Y343F-T25L-T231V as the best starting point, catalyzing the reaction in overall 44% yield with 36% of the C8 adduct being formed and 8% combined yield of the C4 and C5 adducts. One further round of evolution on GluER-T36A-Y343F-T25L-T231V identified GluER-T36A-Y343F-T25L-T231V-F343W as the optimal enzyme and almost doubled the yield of the C8 adduct (65% yield of the C8 adduct, single regioisomer). During these studies, we found by lowering the pH of the buffer to pH 6 GluER-T36A-Y343F furnishes the C5 product in slight majority (46% yield, [0.7:1 C5 : (C4+C8)], making it a promising starting point for engineering. GluER-T36A-Y343F-T25L-T231V-F343W can also alkylate electron-deficient pyridines and pyrazines at the C3 position.

To conclude, we have established a complementary method to traditional arene alkylations by engineering biocatalysts that can control the site selectivity of arene alkylation through the formation of a ternary charge transfer complex between the radical precursor, the arene and the FMN_hq._ Using directed evolution, we engineered a mechanistically promiscuous ERED to favor one mechanism to access an enzyme able to selectively alkylate the C4 position of indole. We also showed engineering can alter the energetics of the charge transfer state to allow ground state electron transfer. We have engineered an enzyme proficient in adding electrophilic radicals into electron-deficient heterocycles like quinoline, enabling the addition of the radical at the C8 position without directing groups or pre functionalization of quinoline. This study demonstrates the ability of directed evolution to tune regioselectivity for arene alkylation and expands the synthetic toolbox available to EREDs. Further engineering efforts could afford enzymes to alkylate arenes at any conceivable position. Additionally, it showcases the ability of EREDs to control radical intermediates to achieve selectivities unparalleled by small molecule methods.

## Figures and Tables

**Figure 1:**
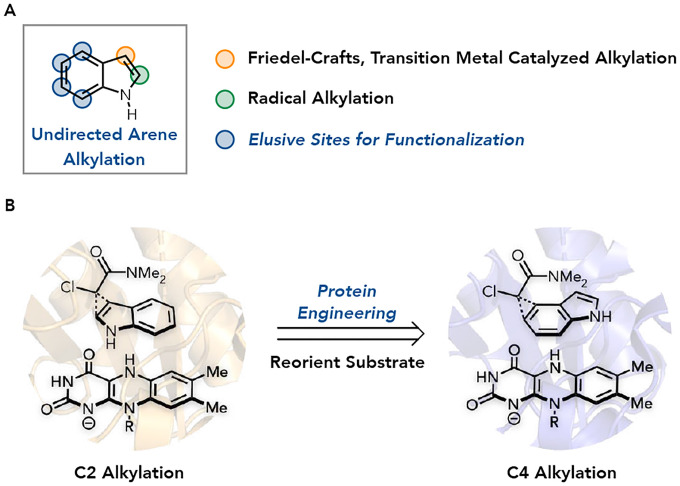
Arene Alkylation Selectivity a. Traditional site selectivity and elusive sites for the functionalization of indole. b. Proposed photoenzymatic regioselective alkylation of indole.

**Figure 2:**
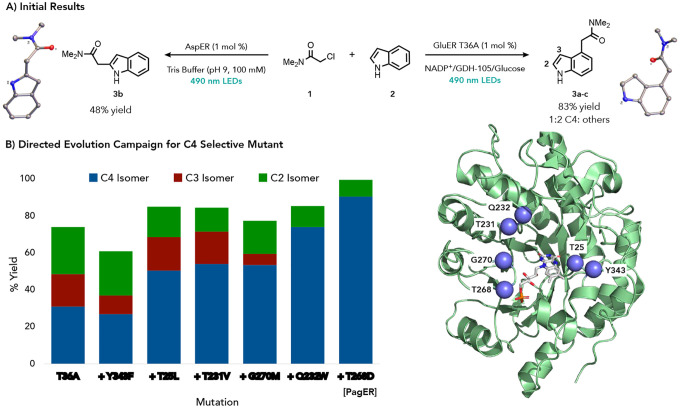
Initial Results and Evolution Campaign. a. Initial results for the biocatalyst-controlled alkylation of indole. b. Results from the evolution campaign. Sites mutated are shown in blue, Reaction conditions: *α*-chloroamide (2 mg, 20 *μ*mol), 3.5 equiv. of arene (70 *μ*mol), 1 mol% ERED, 5 mol% NADP^+^, 1 mg GDH and 6 equiv. of glucose (120 *μ*mol) was irradiated with Cyan LEDS for 24 hours. For AspER reactions were run without NADP^+^, GDH or glucose. Reactions were run in duplicate and yield and regioisomeric ratio was determined by NMR using 1,3,5-trimethoxybenzene as an internal standard.

**Figure 3.**
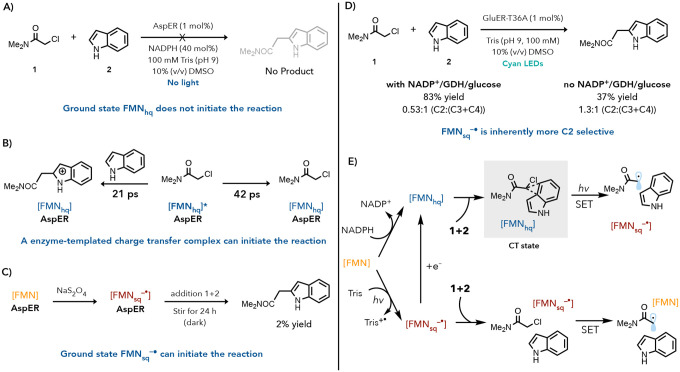
Mechanistic Studies for the initiating species in AspER a. AspER CT state studies b. Ground state hydroquinone is not the initiating species. c. Ground state semiquinone can initate radical formation. d. The ground state semiquinone pathway is more C2 selective. e. Mechanistic proposal for the divergent radical initiation pathways

**Figure 4.**
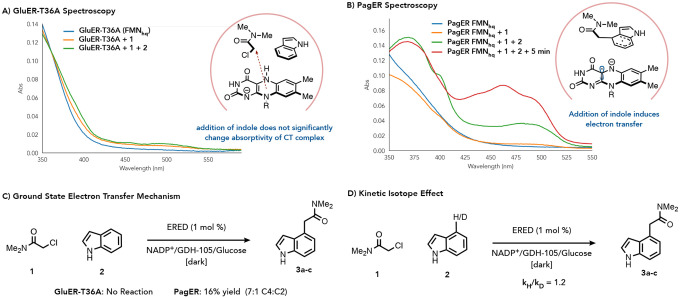
Charge Transfer Complex Studies Reveal Ground State Reactivity was Engineered. a. Charge transfer studies with GluER T36A. b. Charge transfer studies with PagER show ground state electron transfer occurs. c. Engineering of GluER T36A allows for the ground state alkylation of indole. d. KIE studies show the rate limiting step is oxidation of the indoyl radical.

**Figure 3.**
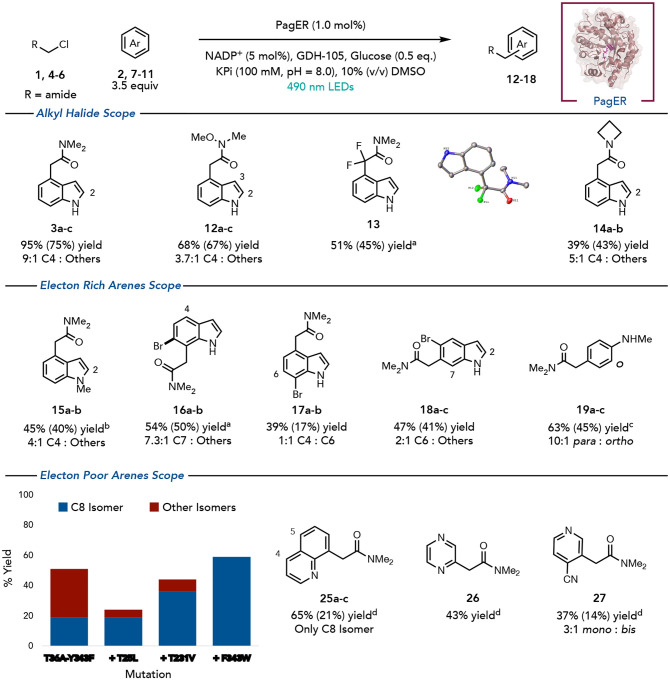
Scope of the biocatalytic alkylation of indole-like arenes using PagER. Reaction conditions: *α*-chloroamide (2 mg, 20 *μ*mol), 3.5 equiv. of arene (70 *μ*mol), 1 mol% PagER, 5 mol% NADP^+^, 1 mg GDH and 0.5 equiv of glucose (10 *μ*mol) was irradiated with Cyan LEDS for 24 hours. Reactions were run in duplicate and yield and regioisomeric ratio was determined by NMR using 1,3,5-trimethoxybenzene as standard. Reactions were scaled up to 200 *μ*mol for isolation and the isolated yields are reported in parantheses. The position of the alkylation in the minor regioisomers is labeled. ^a^with 1.5 mol% enzyme ^b^with GluER T36A-Y343F-T25L-T231V G270M-Q232W ^c^ with GluER T36A-Y343F-T25L. ^d^Reaction conditions: *α*-chloroamide (2 mg, 20 *μ*mol), 3.5 equiv. of arene (70 *μ*mol), 1 mol% GluER T36A-Y343F-T25L-T231V-F343W, 5 mol% NADP^+^, 1 mg GDH and 0.5 equiv of glucose (10 *μ*mol) was irradiated with Cyan LEDS for 24 hours. Reactions were run in duplicate and yield and regioisomeric ratio was determined by NMR using 1,3,5-trimethoxybenzene as standard. Isolated yields are in parantheses. The position of the alkylation for the minor regioisomers is labeled.

## Data Availability

The data that support the findings in this study are available from the corresponding author upon reasonable request. Crystallographic models and structure factors have been deposited in the Protein Data Bank with accession number 8FW1 for PagER. CCDC 2237627–2237629 contains the supplementary crystallographic data for this paper. These data can be obtained free of charge via www.ccdc.cam.ac.uk/data_request/cif, or by emailing data_request@ccdc.cam.ac.uk, or by contacting The Cambridge Crystallographic Data Centre, 12 Union Road, Cambridge CB2 1EZ, UK; fax: +44 1223 336033
